# Fentanyl Inhibits Air Puff-Evoked Sensory Information Processing in Mouse Cerebellar Neurons Recorded *in vivo*

**DOI:** 10.3389/fnsys.2020.00051

**Published:** 2020-08-04

**Authors:** He-Min Yang, Li-Jie Zhan, Xue-Qin Lin, Chun-Ping Chu, De-Lai Qiu, Yan Lan

**Affiliations:** ^1^Brain Science Research Center, Yanbian University, Yanji City, China; ^2^Department of Physiology and Pathophysiology, College of Medicine, Yanbian University, Yanji City, China

**Keywords:** fentanyl, μ-opioid receptor, cerebellar Purkinje cell, molecular layer interneuron, CTOP, protein kinase A

## Abstract

**Aim**: To examine the effects of fentanyl, a potent mu-opioid receptor (MOR) agonist, on-air puff-evoked responses in Purkinje cells (PCs), and molecular layer interneurons (MLIs) using *in vivo* patch-clamp recordings in anesthetized mice.

**Methods**: Male mice 6–8 weeks-old were anesthetized and fixed on a custom-made stereotaxic frame. The cerebellar surface was exposed and perfused with oxygenated artificial cerebrospinal fluid (ACSF). Patch-clamp recordings in the cell-attached mode were obtained from PCs and MLIs. Facial stimulation by air-puff of the ipsilateral whisker pad was performed through a pressurized injection system. Fentanyl citrate, CTOP, and H-89 dissolved in ACSF were perfused onto the cerebellar surface.

**Results**: Fentanyl significantly inhibited the amplitude and area under the curve (AUC) of sensory stimulation-evoked inhibitory responses in PCs. Although fentanyl did not influence the frequency of simple spikes (SSs), it decreased the pause of SS. The IC_50_ of the fentanyl-induced suppression of the P1 response amplitude was 5.53 μM. The selective MOR antagonist CTOP abolished fentanyl-induced inhibitory responses in PCs. However, the application of CTOP alone increased the amplitude, AUC of P1, and the pause of SS. Notably, fentanyl significantly inhibited the tactile-evoked response of MLIs but did not affect their spontaneous firing. The fentanyl-induced decrease of inhibitory responses in PCs was partially prevented by a PKA inhibitor, H-89.

**Conclusions**: These results suggest that fentanyl binds to MORs in MLIs to reduce GABAergic neurotransmission in MLI-PC projections and one potential mechanism is *via* modulation of the cAMP-PKA pathway.

## Introduction

Fentanyl and fentanyl analogs are currently an emerging public health threat around the world. An increasing death rate has been observed not only in North America but also in Europe, Japan, and Brazil [Rudd et al., 2016; UNODC (United Nations Office on Drugs and Crime), 2017]. Fentanyl is an opioid analgesic that was synthesized by *Paul Janssen in the 1960s* in Belgium and is 50–100 times more potent than morphine. During the treatment of chronic pain, fentanyl tolerance occurs when patients need a higher and/or more frequent amount of the drug to get the desired effects. Therefore, fentanyl is now emerging as one of the leading causes of drug overdose lethality in America (Spencer et al., 2019).

As a synthetic, lipophilic phenylpiperidine opioid agonist, fentanyl has a high affinity for μ-opioid receptors (MORs), which underlie major opioid-related pharmacological effects, including analgesia, sedation, and euphoria (Armenian et al., 2018). MORs are distributed throughout the peripheral and central nervous system (CNS), including the cerebellum. Although previous studies showed that MORs were absent in the rodent cerebellum (Mansour et al., 1988), accumulated evidence using more sensitive methods now demonstrates, beyond doubt, the occurrence of functional MORs in the cerebellum (Mrkusich et al., 2004; Peng et al., 2012). MORs belong to the superfamily of G protein-coupled receptors and produces its effects *via* activation of Gαi/o subunits to decrease intracellular levels of cAMP, inhibit calcium channels, and activate inwardly rectifying potassium (Kir) channels (Suzuki and El-Haddad, 2017). MORs in different brain regions have different densities and display different functional roles (Hwang et al., 2010). MORs in the nucleus accumbens and basolateral amygdala underlie euphoria, the incentive properties of rewarding stimuli, and drug abuse (Wang, 2019). Vaughan and Christie (1997) reported that MORs in the periaqueductal gray (PAG) and raphe magnus may be involved in analgesia by inhibiting the activity of GABAergic neurons. However, the effects of MORs in the cerebellum are poorly understood.

The mammalian cerebellar cortex is composed of molecular layer interneurons (MLIs), Purkinje cells (PCs), granule cells, and Golgi cells (Palay and Chan-Palay, 1974). It receives multiple types of sensory inputs and generates motor-related outputs which include motor coordination, sensory perception, and voluntary movements (Yeganeh-Doost et al., 2011). The PCs are the sole output from the cerebellar cortex to the deep cerebellar nucleus neurons and induce inhibition by releasing γ- aminobutyric acid (GABA). There are two major excitatory inputs to PCs, climbing and mossy fibers, which produce complex spikes (CSs) and simple spikes (SSs), respectively. Basket cells and stellate cells are MLIs receiving excitatory inputs from parallel fibers (PFs) and inhibitory inputs from other interneurons. The output of MLIs produces GABAergic inhibition of PCs (Chan-Palay and Palay, 1971; Chu et al., 2011a). The information of tactile stimulation travels past the trigeminal ganglion and continues along the central branch to form synapses in the trigeminal nuclei of the brainstem, and the trigeminal nuclei convey the information to the cerebellar cortex *via* the mossy fibers and the climbing fibers (Bower and Woolston, 1983). We showed previously that facial sensory stimulation of the ipsilateral whisker pad induced GABA-mediated inhibition of PCs by triggering rapid excitation in MLIs and inhibiting the activity of PCs in the Crus II in the cerebellar cortex (Chu et al., 2011b, 2012).

Numerous studies have shown that MORs influence GABAergic inhibitory synaptic transmission (Griffioen et al., 2004; Miura et al., 2008). Therefore, we selected fentanyl as a MOR agonist to investigate its effects on the air puff-evoked responses in cerebellar PCs and MLIs using *in vivo* electrophysiological recordings and pharmacological methods.

## Materials and Methods

### Anesthesia and Surgical Procedures

Male ICR mice [SYXK (Ji) 2007-0011], 6–8 weeks-old, were used for these experiments. They were conducted following protocols approved by the U.S. National Institutes of Health policy. As described previously (Chu et al., 2011a,b), mice were anesthetized with urethane (1.3 g/kg body weight, i.p.) and fixed on a custom-made stereotaxic frame. A 1–1.5 mm opening was drilled on the skull to expose the Crus II of the cerebellar surface, and a watertight chamber was placed on the cerebellar surface to perfuse artificial cerebrospinal fluid (ACSF). Oxygenated ACSF (NaCl 125 mM, KCl 3 mM, MgSO_4_ 1 mM, CaCl_2_ 2 mM, NaH_2_PO_4_ 1 mM, NaHCO_3_ 25 mM, and D-glucose 10 mM) was constantly perfused on the cerebellar surface with a peristaltic pump (Gilson Minipulse 3; Villiers, Le Bel, France) at 0.4 ml/min and animal’s body temperature was maintained at 37.0 ± 0.2°C.

### Electrophysiological Recordings and Facial Stimulation

Patch-clamp recordings in the cell-attached mode were obtained from PCs and MLIs using an Axopatch-200B amplifier (Molecular Devices, Foster City, CA, USA). The recorded signals were acquired through a Digidata 1440 series analog-to-digital interface on a personal computer. The recording electrodes (3–5 MΩ) were filled with ACSF. A 12-gauge stainless-steel tube was used to perform facial stimulation by air-puff (60 ms, 60 psi) of the ipsilateral whisker pad through a pressurized injection system (Picospritzer^®^III). A personal computer was used to control the air-puff stimulations, delivered at 0.05 Hz *via* a Master 8 controller (A.M.P.I.). The Clampfit 10.4 software (Molecular Devices) was used to analyze the electrophysiological data.

### Drug Administration

The following pharmacological agents were used: fentanyl citrate injection (Yichang Renfu pharmaceutical Company Limited, Hubei, China); CTOP (Bio-Techne China Company Limited, Shanghai, China); H89 (Sigma–Aldrich, Shanghai, China). All chemicals were perfused onto the cerebellar surface (0.4 ml/min) in ACSF.

### Statistical Analysis

All results are shown as mean ± SEM. Statistical significance was estimated with student’s paired *t*-tests between the control and experimental groups using SPSS software (Chicago, IL, USA).

## Results

### Fentanyl Inhibits Air Puff-Evoked Inhibitory Responses in Cerebellar PCs

**Figure 1:**
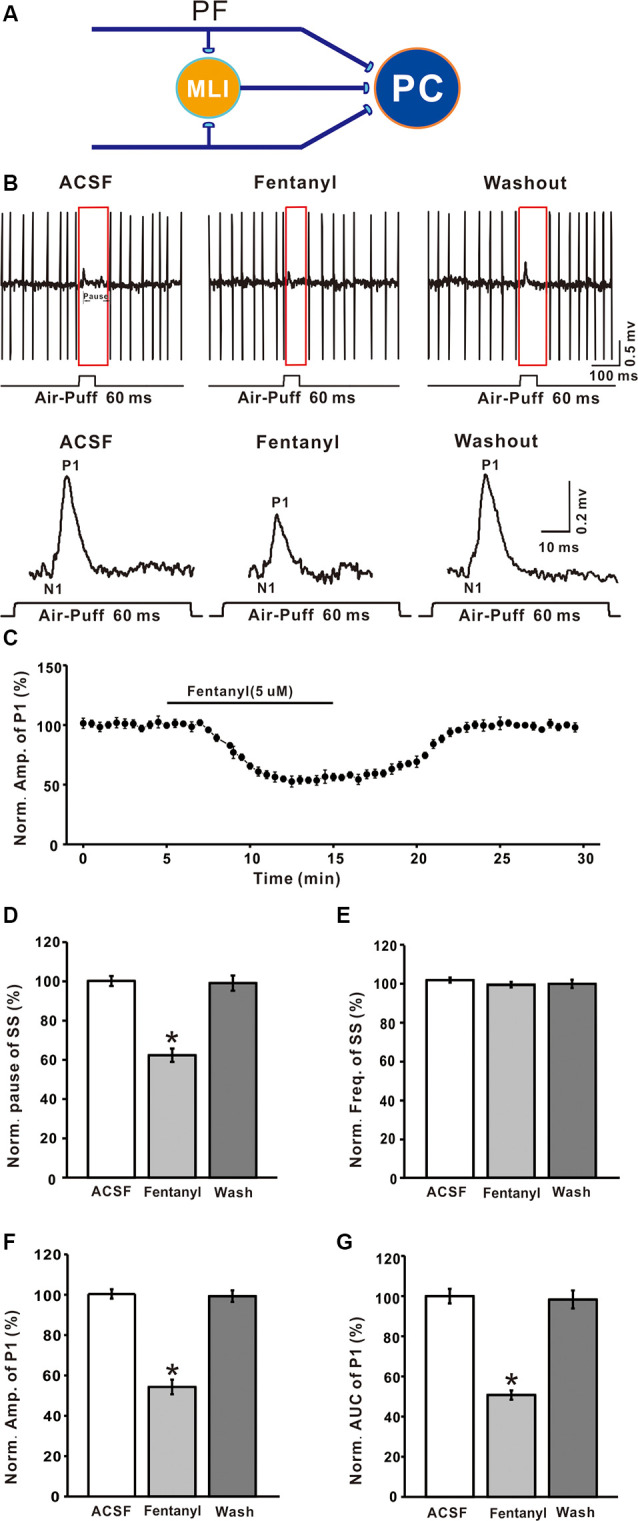
Effects of fentanyl on-air puff-evoked responses in cerebellar Purkinje cells (PCs). **(A)** Excitatory inputs coming from parallel fibers (PFs) and inhibitory inputs coming from the molecular layer interneurons (MLIs) innervate PCs. **(B)** Upper, representative cell-attached recording from a PC showing responses to air-puff stimulation (60 ms, 60 psi) in artificial cerebrospinal fluid (ACSF), fentanyl (5 μM) and washout; lower, enlarged traces from the upper panel showing the effect of fentanyl on sensory-evoked responses. **(C)** Summary of data (*n* = 6) showing the time course of 5 μM fentanyl-induced changes in the amplitude of P1. **(D,E)** Pooled data showing the normalized pause **(D)** and frequency **(E)** of a simple spike (SS) in ACSF, fentanyl (5 μM), and washout. **(F,G)** The bar graph shows the effects of fentanyl (5 μM) on the normalized amplitude **(E)** and area under the curve (AUC, **G**) of P1. *P < 0.05 vs. ACSF group.

PCs are distinguished by the occurrence of SSs with regular spontaneous firing activity, as well as CSs during cell-attached recordings. As in our previous study Chu et al., 2011a, air-puff stimulation (10 ms; 60 psi) evoked responses in PCs are characterized by a negative component (N1), which represents the activity of PFs, followed by a positive component (P1), which represents the air puff-evoked inhibitory response in MLIs-PCs, followed by a pause in SS firing (Figures 1A,B). In this study, application of fentanyl (5 μM) on the cerebellar surface for 10 min did not significantly influence the latency of the response (ACSF: 14.35 ± 0.36 ms; fentanyl: 14.31 ± 0.27 ms, *P* > 0.05; *n* = 6) and it had no effect on the frequency of SS (ACSF: 101.92 ± 1.27% normalized to baseline, *n* = 6; fentanyl: 99.01 ± 1.42%, *n* = 6; *P* > 0.05; Figure 1E). The SS pause was decreased to 62.35 ± 3.41% of the normalized baseline (100.21 ± 2.53%; *n* = 6; *P* < 0.05; Figure 1D). Also, at this concentration, fentanyl induced a reduction in the amplitude of P1 in a time-dependent manner (Figures 1B,C). The amplitude of P1 was significantly depressed by perfusion of fentanyl (fentanyl: 54.33 ± 3.63% normalized to baseline, *n* = 6; ACSF: 100.38 ± 2.30%, *n* = 6; *P* < 0.001; Figure 1F). In addition, the normalized value of the area under the curve (AUC) of P1 was 50.75 ± 3.65% of baseline (100.01 ± 3.65%, *n* = 6; *P* < 0.001; Figure 1G).

**Figure 2:**
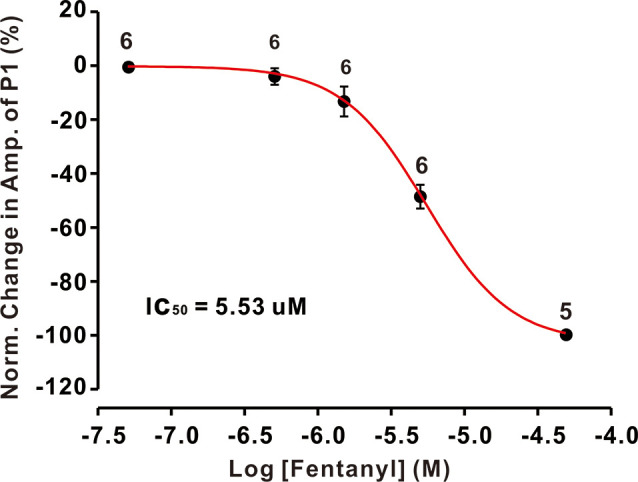
The concentration-response curve shows the fentanyl-induced decrease in the amplitude of P1. The IC_50_ value obtained from the curve was 5.53 μM. The number of PCs examined at each concentration is indicated near the respective bars.

The inhibition of facial stimulation-evoked field potential response induced by fentanyl recovered to baseline after a 20 min washout period. We then investigated the dose-dependency of the fentanyl-induced inhibitory effects on P1. In Figure 2, the lowest effective dose was 500 nM, which decreased P1 to 3.68 ± 1.24% of baseline (*P* < 0.05; *n* = 6), and its IC_50_ was 5.53 μM. When the concentration of fentanyl was increased to 50 μM, the response of the facial stimulation-evoked field potential disappeared. These results indicate that fentanyl, a selective and efficient MOR agonist, dose-dependently inhibited several parameters of the air puff-evoked MLIs-PCs inhibitory responses in the cerebellar PCs layer.

### Blocking MORs Abolished Fentanyl-Induced Suppression of Air Puff-Evoked Inhibitory Responses in Cerebellar PCs

**Figure 3:**
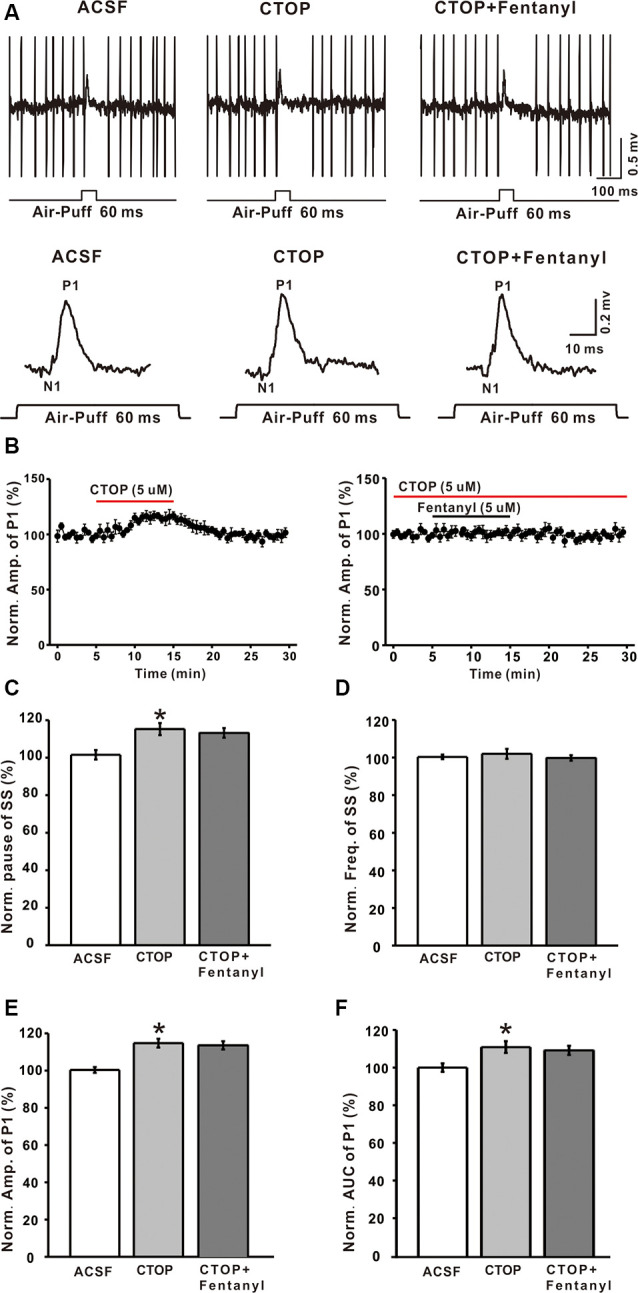
The mu-opioid receptor (MOR) antagonist CTOP abolishes fentanyl-mediated inhibition of facial stimulus-evoked responses in PCs. **(A)** Upper, example responses of a PC to air-puff stimulation (60 ms, 60 psi) in ACSF, CTOP, and CTOP (5 μM) + fentanyl (5 μM); lower, enlarged traces from the upper panel showing the effects of ACSF, CTOP (5 μM) and CTOP (5 μM) + fentanyl (5 μM) on sensory-evoked responses. **(B)** Left, summarized results showing the time course of 5 μM CTOP-induced changes in the amplitude of P1 (*n* = 6); Right, pooled data (*n* = 6) showing the time course of fentanyl-induced changes in the amplitude of P1 in the presence of CTOP. **(C,D)** Summarized data showing the normalized pause **(C)** and frequency **(D)** of SSs in ACSF, CTOP (5 μM), and CTOP (5 μM) + fentanyl (5 μM). **(E,F)** The bar graph shows the effects of CTOP (5 μM) and CTOP (5 μM) + fentanyl (5 μM) induced changes on the normalized amplitude **(E)** and AUC **(F)** of P1. *P < 0.05 vs. ACSF group.

Although it has been reported that MORs exist in the cerebellum, the maximal number of MORs is still less than in various other brain structures, but the function of MORs in the cerebellum is still not clear. To examine whether the fentanyl-induced inhibition of GABAergic response was indeed mediated by MORs, we investigated the effects of fentanyl on P1 responses in the presence of CTOP, a selective MORs antagonist. Perfusion of CTOP alone (5 μM) produced a significant increase in the amplitude of P1 (CTOP: 114.73 ± 2.37% of baseline, *n* = 6, *P* < 0.05; Figures 3A,B,E), the pause of SS (CTOP: 115.23 ± 3.21% of baseline, *n* = 6, *P* < 0.05; Figure 3C) and the AUC of P1 (CTOP: 110.89 ± 3.12% of baseline, *n* = 6, *P* < 0.05; Figure 3F). However, CTOP alone or in conjunction with fentanyl had no effect on the pause of SS (CTOP + fentanyl: 113.21 ± 2.56% of baseline, *P* > 0.05 compared to CTOP, *n* = 6; Figure 3C), the amplitude of P1 (CTOP + fentanyl: 112.07 ± 2.16% of baseline, *P* > 0.05 compared to CTOP, *n* = 6; Figures 3A,B,E), and AUC (CTOP + fentanyl: 109.15 ± 2.43% of baseline, *P* > 0.05 compared to CTOP, *n* = 6; Figure 3F). Further, fentanyl had no effect on the frequency of SS (ACSF: 99.99 ± 2.25% of baseline, *n* = 6; CTOP: 101.98 ± 2.64% of baseline, *P* > 0.05 compared to ACSF, *n* = 6; CTOP + fentanyl: 99.78 ± 1.42% of baseline, *P* > 0.05 compared to CTOP, *n* = 6; Figure 3D). These results indicate that endogenous opiates exist in the MLIs-PCs synapses and that there is an endogenous opiate inhibition of GABAergic tone in MLIs-PCs information transmission. Also, CTOP prevented fentanyl-mediated inhibitory responses in cerebellar PCs. These results suggested that the inhibition induced by fentanyl is due to the activation of MORs.

### Effects of Fentanyl on Air Puff-Evoked Responses in MLIs

**Figure 4:**
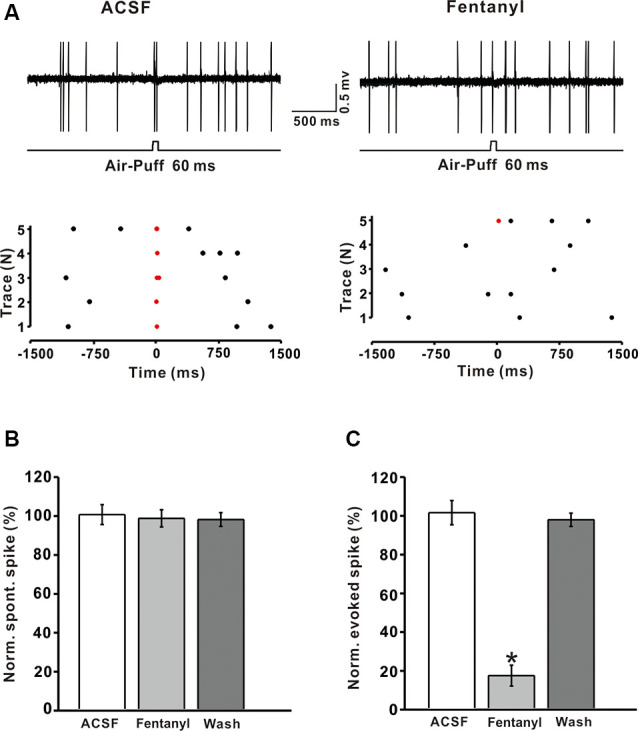
Fentanyl depresses air-puff stimulation-evoked responses in cerebellar molecular layer interneurons (MLIs).** (A)** Upper, an example of cell-attached recording from an MLI showing responses to air-puff stimulation (60 ms, 60 psi) in ACSF, and fentanyl (5 μM); lower, raster plot of spike events originating from traces in the upper panel. **(B)** Summarized results showing the normalized frequency of spontaneous firing in ACSF, fentanyl (5 μM, *n* = 6). **(C)** Summarized results showing the normalized frequency of tactile-evoked firing in ACSF and fentanyl (5 μM, *n* = 6). *P < 0.05 vs. ACSF group.

Previous immunohistochemical studies suggested that MORs exist abundantly in GABAergic neurons in the CNS (Mrkusich et al., 2004). To examine whether the decrease of fentanyl-induced GABAergic response occurred *via* inhibiting the activity of MLIs, we investigated the effects of fentanyl on the spontaneous and tactile-evoked spike firing of MLIs. Using the cell-attached recording technique, we found that air-puff stimulation of the ipsilateral whisker pad evoked spike firing in cerebellar MLIs, consistent with our previous report (Chu et al., 2012; Figure 4A). Application of fentanyl (5 μM) for 10 min did not change the spontaneous firing frequency of MLIs but inhibited facial stimulation-evoked firing (Figures 4A,B). After perfusion of fentanyl (5 μM), the normalized frequency of spontaneous spike firing was 99.05 ± 4.41% of baseline (ACSF: 100.00 ± 5.12%; *P* > 0.05; *n* = 6; Figure 4B). The normalized frequency of tactile-evoked firing was decreased by 16.67 ± 5.35% of baseline (ACSF: 100.21 ± 6.23%; *P* < 0.001; *n* = 6; Figure 4C). These results indicate that MLIs express MORs, and the application of fentanyl inhibits tactile-evoked firing in cerebellar MLIs, resulting in a decrease of tactile-evoked inhibitory responses in PCs.

### cAMP-PKA Signaling Pathway Participates in the Suppression of Air Puff-Evoked Inhibitory Responses in PCs

**Figure 5:**
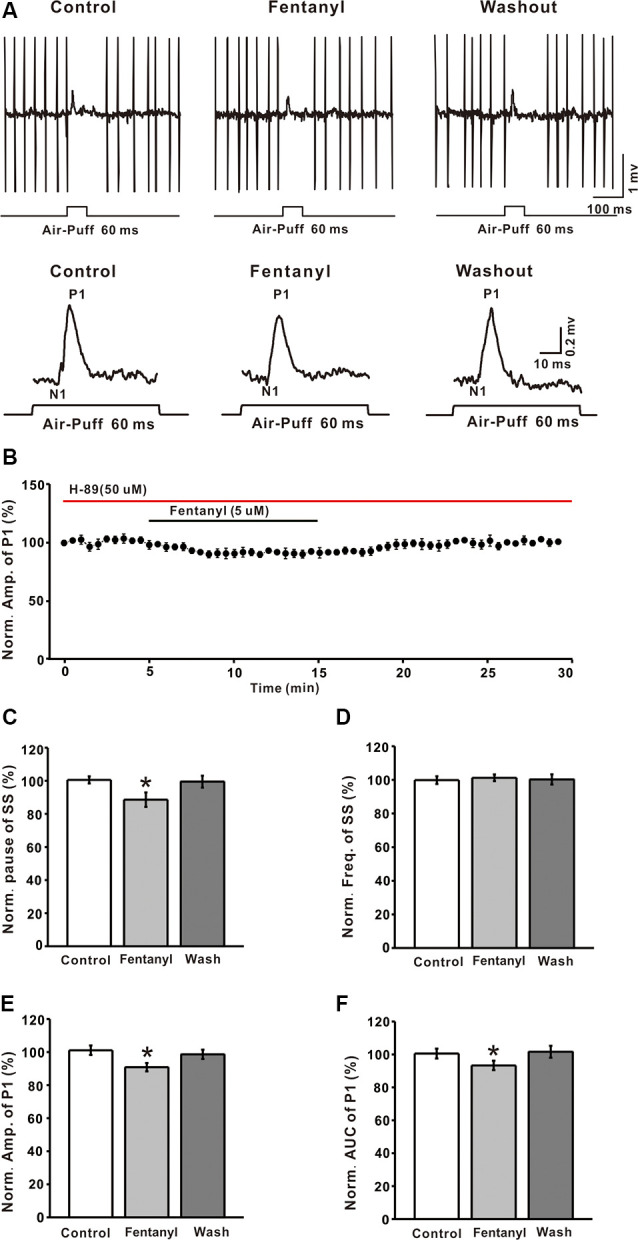
The protein kinase A blocker, H-89 partially prevented the fentanyl-induced inhibition of P1 in cerebellar PCs. **(A)** Upper, an example of cell-attached recording from a PC showing responses to facial stimulation (60 ms, 60 psi) in control fentanyl (5 μM) and washout in the presence of H-89; lower, enlarged image from the upper panel showing the effects of fentanyl on air-puff stimulation-evoked responses in the presence of H-89. **(B)** Summarized results showing the time course of 5 μM fentanyl-induced changes in the amplitude of P1 in the presence of H-89 (*n* = 6). **(C,D)** Summarized data showing the normalized pause **(C)** and frequency **(D)** of simple spikes (SSs) in control, fentanyl (5 μM), and washout in the presence of H-89. **(E,F)** Bar graph showing the effects of fentanyl (5 μM) on the normalized amplitude **(E)** and area under the curve (AUC) **(F)** of P1 in the presence of H-89. *P < 0.05 vs. control group.

It is well known that MORs couple to the G_αi/o_ subunits, reduces the content of cAMP, and depresses the activity of PKA. To explore the mechanism of fentanyl-induced inhibition in MLIs-PCs GABAergic responses to facial stimulation, we applied the selective PKA inhibitor: H-89 (5 μM). As shown in Figures 5A,B, H– 89 partially abrogated the fentanyl-induced inhibition of facial stimulation-evoked GABAergic responses in PCs. In the presence of H-89 (50 μM), the fentanyl-induced decrease in the amplitude of P1 was 90.86 ± 2.56% (*n* = 6) of control, 101.10 ± 2.85% (*n* = 6, *P* = 0.027; Figure 5E). The fentanyl-induced decrease in the AUC of P1 was also partly suppressed by H-89, and the inhibition in the AUC of P1 was 93.32 ± 2.86% of control (100.50 ± 2.99% of baseline, *n* = 6; *P* < 0.05; Figure 5F), which was significantly smaller than the inhibition induced by fentanyl alone. However, in the presence of H-89, fentanyl had no effect on the frequency of SS (control: 99.87 ± 2.31% *n* = 6; fentanyl: 101.24 ± 1.96% *n* = 6; *P* > 0.05; Figure 5D), but partially abolished the decrease of the pause of SS (control: 100.56 ± 2.14% *n* = 6; fentanyl: 88.63 ± 4.36% *P* > 0.05, *n* = 6; Figure 5C). In order to further confirm if PKA signaling pathway participate the regulation of fentanyl-induced inhibition in MLIs-PCs GABAergic responses to facial stimulation, we applied the AC agonist, forskolin. As shown in Supplementary Figure S1A, forskolin reserved the fentanyl-induced inhibition of facial stimulation-evoked GABAergic responses in PCs. According to these data, inhibition of the PKA pathway significantly suppressed the fentanyl-induced decreases in the amplitude, AUC, and the pause of SS. The results also indicate that fentanyl decreased facial stimulation-evoked MLIs-PCs GABAergic synaptic responses partially by affecting the cAMP-PKA signaling pathway.

## Discussion

In this study, we assessed the effects of fentanyl on the sensory stimulation-evoked GABAergic component of PCs response. We found that fentanyl significantly decreased the amplitude and AUC of the PCs response to facial stimulation. It is interesting that the application of CTOP alone significantly increased the amplitude and AUC of P1. The results from these experiments demonstrated that MORs distribute in the MLIs-PCs synapses, dose-dependently inhibit several parameters of the sensory stimulation-evoked MLIs-PCs synaptic responses in the PCs layer of the cerebellum and that there is endogenous opiate inhibition of MLIs-PCs GABAergic transmission. Notably, fentanyl significantly inhibited sensory stimulation-evoked responses of MLIs but did not change the spontaneous firing rate. These results show that fentanyl suppresses the sensory stimulation-evoked responses by inhibiting the activity of MLIs. Importantly, the fentanyl-induced decrease of P1 was partially prevented by the administration of a PKA inhibitor, suggesting that the cAMP-PKA signaling pathway participates in the suppression of air puff-evoked inhibitory responses in PCs.

### Fentanyl Decreases Air Puff-Evoked MLIs-PCs GABAergic Synaptic Responses by Inhibiting the Activity of MLIs to Tactile Stimulation

Fentanyl is a synthetic MORs prescription drug that acts at the opioid receptors to regulate pain and emotions. Owing to the limitations of experimental methods, scientists once thought that MORs were absent in the rodent cerebellum. However, growing evidence suggests that MORs are indeed present in the cerebellum. It has been reported that the MOR protein and MOR mRNA are expressed in the PC layer, the granular layer, and the molecular layer of the adult and postnatal rat cerebellum (Mrkusich et al., 2004). Furthermore, in-depth research of cerebellar function shows that the cerebellum is not only a motor structure, it also has critical non-motor functions involved in normal and disease states such as schizophrenia, learning, and memory, analgesia, and addiction (Strick et al., 2009). For example, Carta et al. (2019) reported that mice express a strong preference for the reward quadrant in conditioned place preference training by optogenetic activation of the cerebellar-VTA projections. Clinical research found that damage to the posterior cerebellar hemispheres disrupts affective pain processing and endogenous pain modulation, resulting in decreased pain tolerance to suprathreshold noxious stimuli (Silva et al., 2019).

In the cerebellar cortex, the dendrites of stellate-type MLIs act on PCs dendrites, whereas the axons of basket-type MLIs directly inhibit the soma and initial segment of PCs (Chu et al., 2012; He et al., 2015). The *in situ* results demonstrate that fentanyl decreases the amplitude and AUC of tactile stimulation-evoked GABAergic responses in the cerebellar PC layer. The inhibition was blocked by CTOP, a selective antagonist of MORs, suggesting its action *via* MORs to modulate GABAergic synaptic transmission. However, the application of CTOP alone increased the amplitude and AUC of sensory stimulation-evoked inhibitory responses, indicating that an endogenous opioid participates in the inhibition of GABAergic activity in MLIs-PCs synapses. Acute activation of MORs tonically inhibits GABAergic transmission in the central amygdala (CeA) and MORs reduce presynaptic GABA release to decrease GABAergic transmission in most CeA neurons (Bajo et al., 2014). Indeed, it is well documented that fentanyl affects the excitability of neurons by suppressing the presynaptic neurotransmitter release of GABA elsewhere in the brain, including the lateral habenula (Margolis and Fields, 2016), hippocampus (Alreja et al., 2000), forebrain (Ben Hamida et al., 2019) and CeA (Blaesse et al., 2015). Besides, both endogenous enkephalin and endorphins act on presynaptic GABAergic neurons in the hippocampus and the amygdala (Bali et al., 2015). Our results demonstrate that the fentanyl-induced inhibition in the amplitude and AUC of P1 might be due to the activation of MORs, inducing a decrease of sensory-evoked responses in MLIs, which leads to less GABA released by MLIs in response to the stimulation.

### Fentanyl-Mediated Suppression of Air Puff-Evoked Inhibitory Responses in Cerebellar PCs Is Dependent on the cAMP-PKA Signaling Pathway

The exact mechanism by which fentanyl inhibits air puff-induced GABAergic synaptic responses in PCs is unknown. However, fentanyl is known to couple MORs, and the MOR is a G protein-coupled receptor that acts on Gi/o alpha-subunit proteins to reduce the content of cAMP, which in turn depresses PKA activity (Scavone et al., 2013; Kimura and Haji, 2014). In this study, we explored the role of the cAMP-PKA signaling pathway in fentanyl-induced inhibition of cerebellar PCs. The results indicated that the fentanyl-induced decrease of air puff-evoked responses in PCs is largely prevented by PKA inhibitors, e.g., H-89, suggesting that fentanyl inhibits air puff-evoked inhibitory responses partially by activating MORs *via* the cAMP-PKA pathway.

PKA is known as a cAMP-dependent protein kinase and plays an essential role in MORs effects. Acute morphine application decreased the frequency of mIPSCs in CeA neurons *via* the Gi/o signaling pathway to inhibit GABA release in CeA (Bajo et al., 2014). It was reported that suppression in the level of intracellular cAMP and the resultant protein kinase A (PKA) inhibition induces the opening of K^+^ channels and/or the closing of voltage-dependent calcium channels, resulting in pre- and postsynaptic inhibition (Miura et al., 2008). Activation of presynaptic MORs inhibited corticostriatal excitatory inputs and was shown to be mediated by the cAMP-PKA signaling pathway (Barral et al., 2003). Likewise, suppression of the PKA pathway in the striatum induced decreases in GABA release *via* activation of MOR (Miura et al., 2007). However, H-89 does not completely reverse fentanyl-induced inhibition of air puff-evoked responses in PCs, therefore, we cannot rule out the possibility that other mechanisms are involved in these changes. One of the reasons is that the effects of cAMP cannot be solely attributed to PKA. On the one hand, cAMP can activate exchange proteins directly, and cAMP-gated ion channels, as downstream effectors, also play a role in cAMP signaling. Besides, many neuromodulators induce their effects through G protein-regulated intracellular signaling pathways in the CNS, and the cAMP-PKA pathway plays an important role in regulating excitability in many types of neurons (Liang et al., 2018). Our results suggest that fentanyl inhibits PKA *via* a Gi/o-mediated pathway, decreasing GABA release from MLIs. Examination of the mechanisms involved in PKA-mediated suppression of fentanyl-induced inhibition of air puff-evoked responses merits further studies.

In summary, the tactile experience through whiskers is the main way to get information from the outside world for rodents. In this study, we use electrophysiology and pharmacological methods to demonstrates that fentanyl acts *via* MORs on cerebellar MLIs to reduce MLIs-PCs GABAergic responses. The fentanyl mediated inhibition of GABAergic responses through the cAMP-PKA pathway may be one mechanism by which the cerebellum participates in the regulation of cerebellar motor and non-motor functions *via* MORs. Of course, the functions of MORs in the cerebellum need to be further studied.

## Data Availability Statement

All datasets presented in this study are included in the article/Supplementary Material.

## Ethics Statement

The animal study was reviewed and approved by Animal Care and Use Committee of Yanbian University.

## Author Contributions

D-LQ and YL conceived and designed the experiments. H-MY, L-JZ and X-QL performed the experiments. X-QL, C-PC, D-LQ, and YL analyzed the data. D-LQ and YL wrote the manuscript.

## Conflict of Interest

The authors declare that the research was conducted in the absence of any commercial or financial relationships that could be construed as a potential conflict of interest.

## Supplementary Material

FIGURE S1 The adenylate cyclase agonist, forskolin reversed the fentanyl-induced inhibition of P1 in cerebellar PCs. **(A)** Upper, example of cell-attached recording from a PC showing responses to facial stimulation (60 ms, 60 psi) in ACSF, fentanyl (5 μM) and fentanyl (5 μM) + forskolin (30 μM); lower, enlarged image from the upper panel showing the effects of fentanyl on air-puff stimulation-evoked responses. **(B)** Summarized results showing the time course of forskolin reversing the fentanyl-induced inhibition of P1 in cerebellar PCs (*n* = 6). **(C,D)** Summarized data showing the normalized pause **(C)** and frequency **(D)** of simple spikes in ACSF, fentanyl (5 μM) and fentanyl (5 μM) + forskolin (30 μM). **(E,F)** Bar graph showing the effects of fentanyl (5 μM) and fentanyl (5 μM) + forskolin (30 μM) on the normalized amplitude **(E)** and area under the curve (AUC, **F**) of P1. **P* < 0.05 vs. fentanyl group.

## Abbreviations

MOR: mu-opioid receptor
MLIs: Molecular layer interneurons
PCs: Purkinje cells
GABA: γ-aminobutyric acid
PF: parallel fiber
SS: simple spike.
