# Supplementary figures and images for: Fentanyl Inhibits Air Puff-Evoked Sensory Information Processing in Mouse Cerebellar Neurons Recorded in vivo
Source: Front Syst Neurosci. 2020 Aug 4;14:51. doi: 10.3389/fnsys.2020.00051 (PMC7417629; doi:10.3389/fnsys.2020.00051)

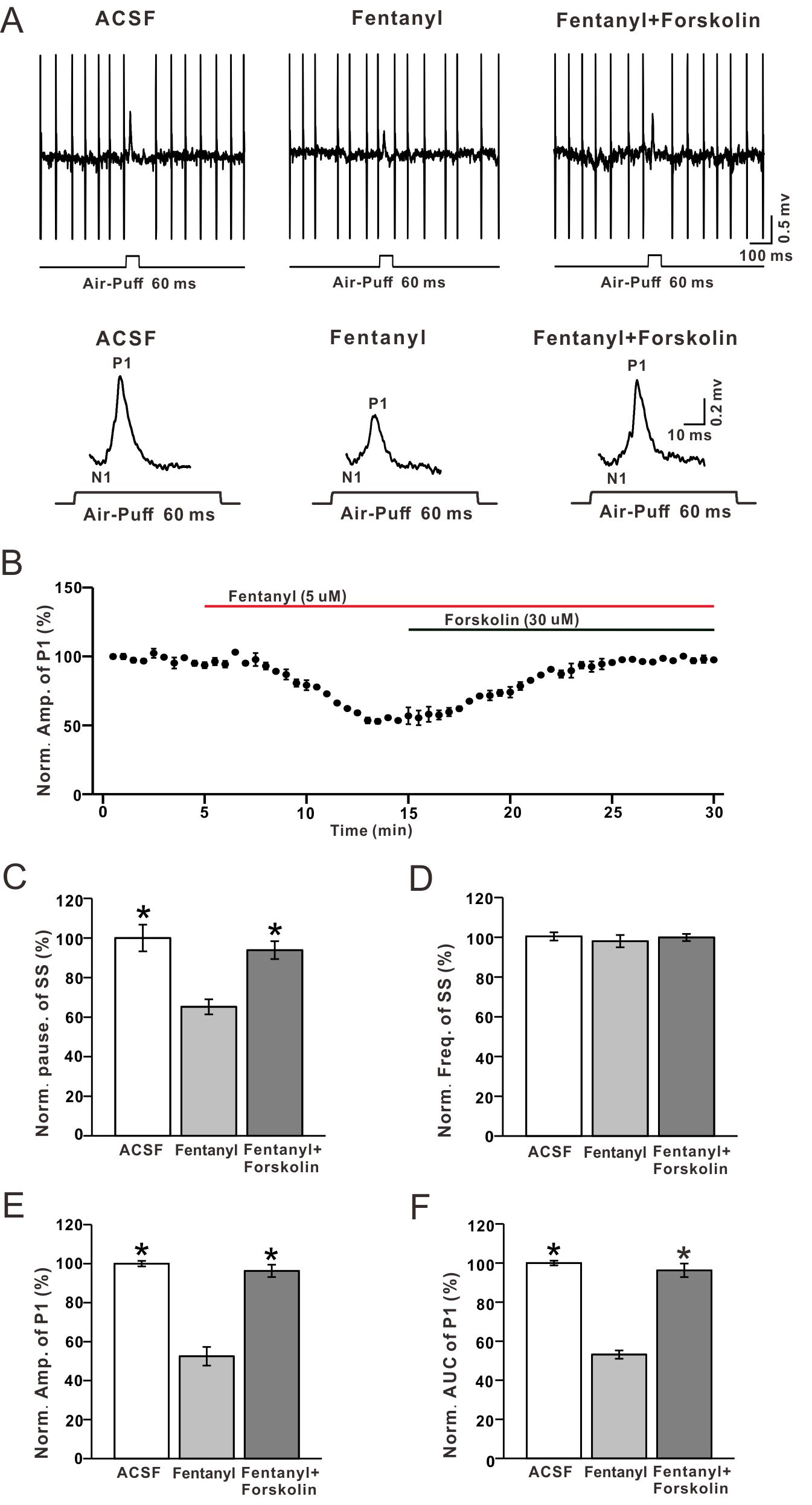

Supplement: FIGURE S1 — The adenylate cyclase agonist, forskolin reversed the fentanyl-induced inhibition of P1 in cerebellar PCs. (A) Upper, example of cell-attached recording from a PC showing responses to facial stimulation (60 ms, 60 psi) in ACSF, fentanyl (5 μM) and fentanyl (5 μM) + forskolin (30 μM); lower, enlarged image from the upper panel showing the effects of fentanyl on air-puff stimulation-evoked responses. (B) Summarized results showing the time course of forskolin reversing the fentanyl-induced inhibition of P1 in cerebellar PCs (n = 6). (C,D) Summarized data showing the normalized pause (C) and frequency (D) of simple spikes in ACSF, fentanyl (5 μM) and fentanyl (5 μM) + forskolin (30 μM). (E,F) Bar graph showing the effects of fentanyl (5 μM) and fentanyl (5 μM) + forskolin (30 μM) on the normalized amplitude (E) and area under the curve (AUC, F) of P1. *P < 0.05 vs. fentanyl group. [file Image_1.JPEG]
